# Pulmonary Mucormycosis Presenting as Non-resolving Pneumonia in an Elderly Patient With Uncontrolled Diabetes Mellitus: A Case Report

**DOI:** 10.7759/cureus.110004

**Published:** 2026-05-31

**Authors:** Thayani Raja, Nirasha Jayathilake, Manil Peiris

**Affiliations:** 1 Internal Medicine, Batticaloa Teaching Hospital, Batticaloa, LKA; 2 Respiratory Medicine, National Hospital, Kandy, Kandy, LKA

**Keywords:** amphotericin b, bronchoscopy, diabetes mellitus, immunocompromised host, invasive fungal infection, non-resolving pneumonia, pulmonary mucormycosis

## Abstract

Pulmonary mucormycosis is an uncommon but highly aggressive opportunistic fungal infection associated with significant morbidity and mortality, particularly in immunocompromised individuals. Early diagnosis remains challenging due to its nonspecific clinical and radiological presentation, often mimicking bacterial pneumonia or other pulmonary infections. We report the case of a 78-year-old woman with poorly controlled type 2 diabetes mellitus who presented with a prolonged history of productive cough, pleuritic chest pain, and intermittent fever. Despite treatment with broad-spectrum antibiotics, her condition failed to improve, prompting further evaluation. Imaging revealed persistent pulmonary consolidation, and bronchoscopy demonstrated necrotic endobronchial lesions. Histopathological examination confirmed the presence of broad, aseptate fungal hyphae, and fungal culture was consistent with mucormycosis. The patient was successfully treated with intravenous liposomal amphotericin B, resulting in significant clinical and radiological improvement. This case highlights the importance of considering invasive fungal infections such as mucormycosis in cases of non-resolving pneumonia, especially in patients with uncontrolled diabetes. Prompt recognition, early initiation of appropriate antifungal therapy, and correction of underlying risk factors are critical in improving patient outcomes.

## Introduction

Mucormycosis is an uncommon yet highly fatal opportunistic fungal infection that predominantly affects individuals with impaired immunity [1,2]. It is caused by fungi of the order Mucorales. Transmission to humans mainly occurs through inhalation of airborne sporangiospores, although ingestion of contaminated substances or direct inoculation through disrupted skin can also occur [2].

A characteristic feature of mucormycosis is its angioinvasive nature, contributing to significant morbidity and mortality [3]. The rhino-cerebral variant is most frequently observed in patients with poorly controlled diabetes mellitus, while pulmonary involvement is less common and is typically encountered in individuals with hematological malignancies or those who have undergone organ transplantation [4].

Definitive diagnosis is established by demonstrating broad, aseptate hyphae on histopathological examination, supported by fungal culture [5]. Management requires a multimodal approach, including prompt surgical debridement of necrotic tissue along with appropriate systemic antifungal therapy [1,6].

## Case presentation

A 78-year-old woman with a known history of poorly controlled type 2 diabetes mellitus, hypertension, and stage G3a chronic kidney disease was transferred from the medical ward to the respiratory unit for further evaluation of a non-resolving pneumonia. She initially presented with a three-month history of productive cough and right-sided pleuritic chest pain. These symptoms were associated with generalized fatigue and intermittent fever. She denied anorexia or weight loss and had no history of tuberculosis exposure. She was a lifelong non-smoker and did not consume alcohol. There was no history of exposure to avian antigens, and she did not report symptoms suggestive of an underlying connective tissue disorder.

On examination, she was afebrile but tachypneic. Chest auscultation revealed bilateral coarse crackles. Her oxygen saturation on room air was 94%. Examination of other systems was unremarkable.

Laboratory investigations demonstrated an elevated erythrocyte sedimentation rate (95 mm in the first hour) and a markedly raised C-reactive protein level (199 mg/dL). Full blood count showed leukocytosis with a white cell count of 13.46 × 10³/µL and a hemoglobin level of 9.5 g/dL. Serum creatinine was elevated at 1.7 mg/dL (baseline serum creatinine of 1.7 mg/dL). Liver function tests, urinalysis, 12-lead electrocardiogram, two-dimensional echocardiography, and coagulation profile were within normal limits (Table 1).

**Table 1:** Laboratory investigation results on admission.

Parameter	Patient Value	Normal Reference Range
Erythrocyte Sedimentation Rate (ESR)	95 mm/1st hour	Adult female: 0-20 mm/hr
C-Reactive Protein (CRP)	199 mg/dL	< 0.5 mg/dL (often <1.0 mg/dL)
White Blood Cell Count (WBC)	13.46 × 10³/µL	4.0-11.0 × 10³/µL
Hemoglobin (Hb)	9.5 g/dL	Female: 12.0-15.0 g/dL
AST (Aspartate Aminotransferase)	35 U/L	10-40 U/L
ALT (Alanine Aminotransferase)	23 U/L	7-56 U/L
Serum Creatinine (S. Cr)	1.7 mg/dL	0.5-1.1 mg/dL (females)
INR (International Normalized Ratio)	1.1	0.8-1.2

Her random blood glucose level on admission was 300 mg/dL, although arterial blood gas analysis showed no evidence of lactic acidosis. Chest radiography revealed heterogeneous opacification in the right upper lobe (Figure 1).

**Figure 1:**
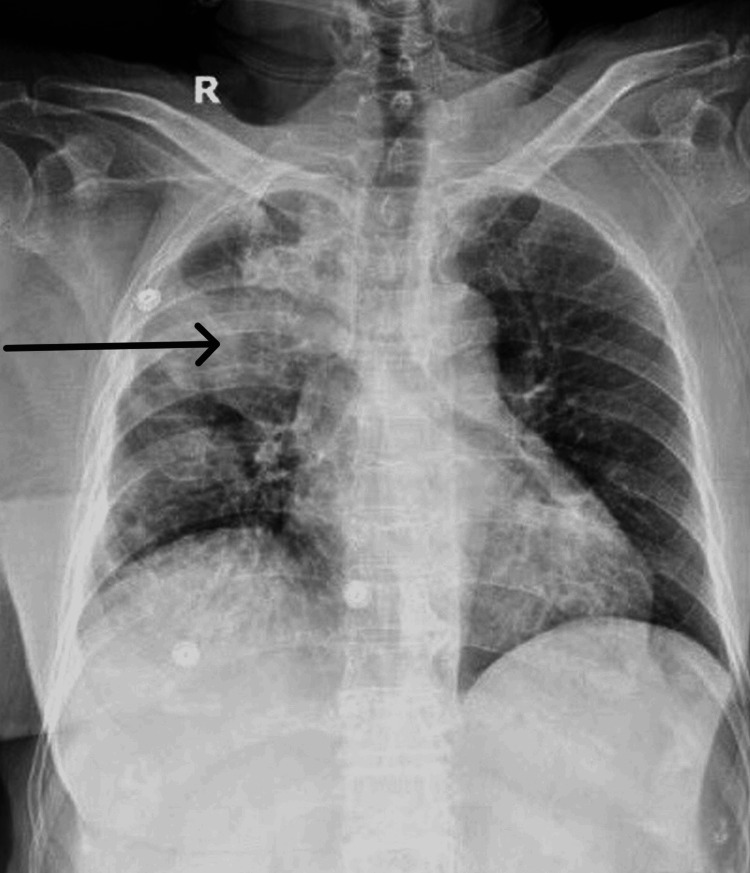
Chest X-ray taken on admission showing consolidation on the right upper lobe (black arrow). Chest X-ray (posterior-anterior view) on admission showing right upper lobe consolidation (indicated by the black arrow).

She was commenced on broad-spectrum antibiotic therapy with intravenous meropenem 1 g every eight hours due to persistent symptoms and failure to respond to other antibiotics. Glycemic control was achieved with insulin therapy. Given the presence of right upper lobe opacification on chest radiograph, she was evaluated for pulmonary tuberculosis and invasive aspergillosis, both of which were negative. Blood and urine cultures showed no microbial growth. Due to the lack of clinical improvement despite appropriate antibiotic therapy for community-acquired pneumonia, a contrast-enhanced computed tomography scan of the chest was performed. This demonstrated bilateral lung consolidations, right-sided empyema, and a subpulmonic pleural effusion, suggestive of a complicated infective process. There was dense consolidation in the right upper lobe with obliteration of the apical segmental bronchus; however, no definite underlying mass lesion was identified (Figure 2).

**Figure 2:**
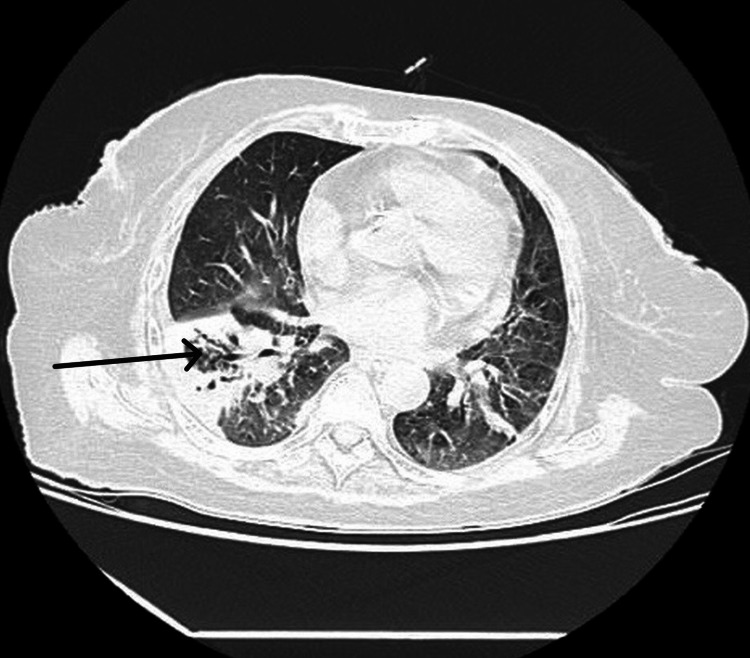
CECT of the chest. Contrast-enhanced computed tomography (CECT) chest (axial view) showing dense consolidation in the right upper lobe with obliteration of the apical segmental bronchus (indicated by the black arrow).

Subsequently, bronchoscopy was performed to obtain diagnostic samples and to exclude alternative etiologies. Bronchoscopy examination revealed obstruction of the anterior and apical segments of the right upper lobe bronchus by a whitish necrotic lesion (Figure 3).

**Figure 3:**
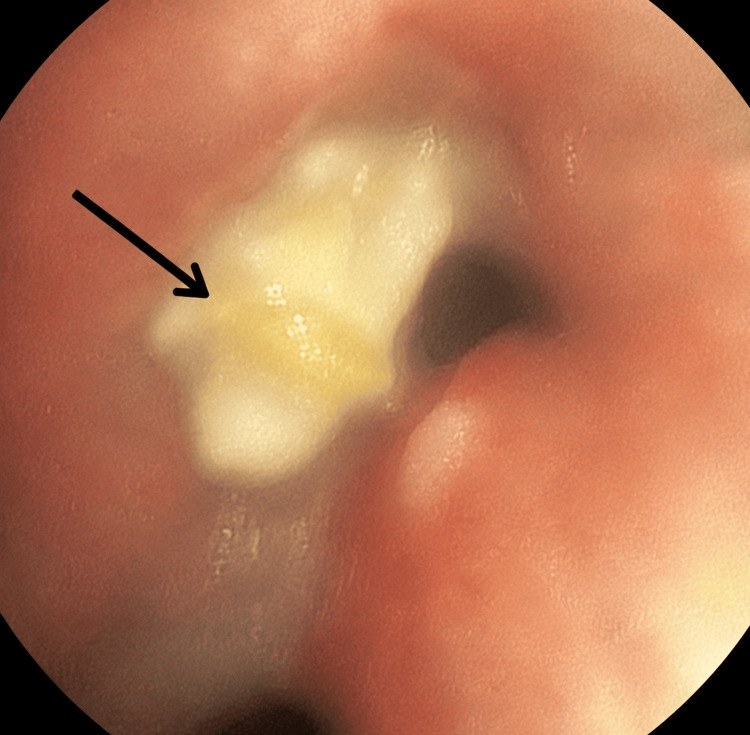
Flexible optic bronchoscopy view. Flexible optic bronchoscopy showing whitish necrotic impaction in the right upper lobe bronchus (indicated by the black arrow).

Tissue samples were obtained for bacterial culture, tuberculosis polymerase chain reaction, fungal studies, and histopathological examination. Tuberculosis PCR was negative. Fungal culture demonstrated blackish growth consistent with mucormycosis. Histopathological analysis showed necrotic tissue with acute inflammatory exudate and numerous broad, aseptate fungal hyphae with spores (Figure 4).

**Figure 4:**
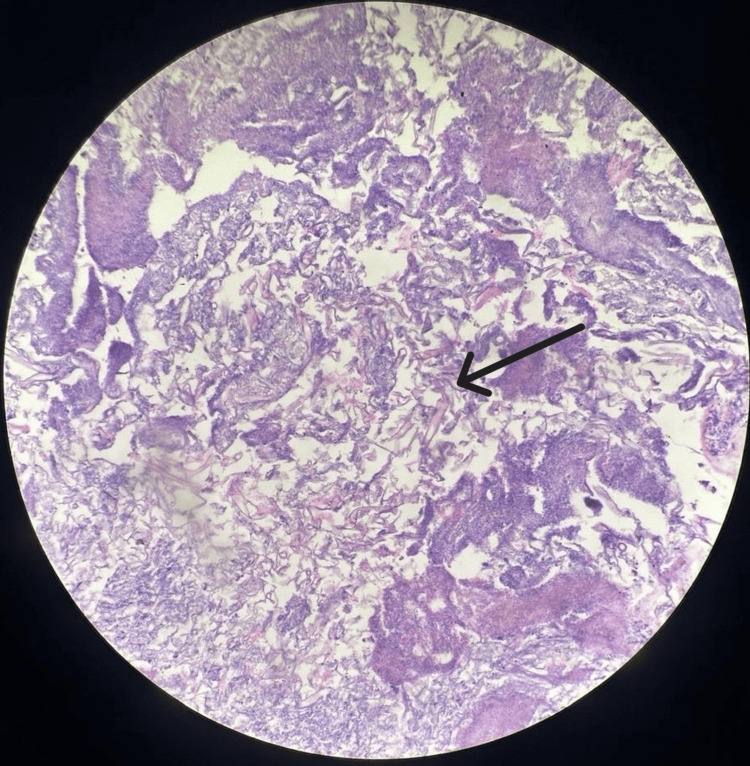
Histopathology findings. Histopathology of endobronchial biopsy (hematoxylin and eosin stain, original magnification ×100) showing necrotic tissue with acute inflammatory exudate and numerous broad, aseptate fungal hyphae (black arrow), suggestive of mucormycosis.

A diagnosis of invasive pulmonary mucormycosis was established. The patient was treated with intravenous liposomal amphotericin B at a dose of 140 mg daily for 22 days. Renal function was closely monitored throughout therapy, and adequate hydration was maintained to minimize the risk of nephrotoxicity. Following initiation of antifungal treatment, there was significant clinical improvement, and a repeat chest radiograph demonstrated marked resolution of the pulmonary infiltrates (Figure 5).

**Figure 5:**
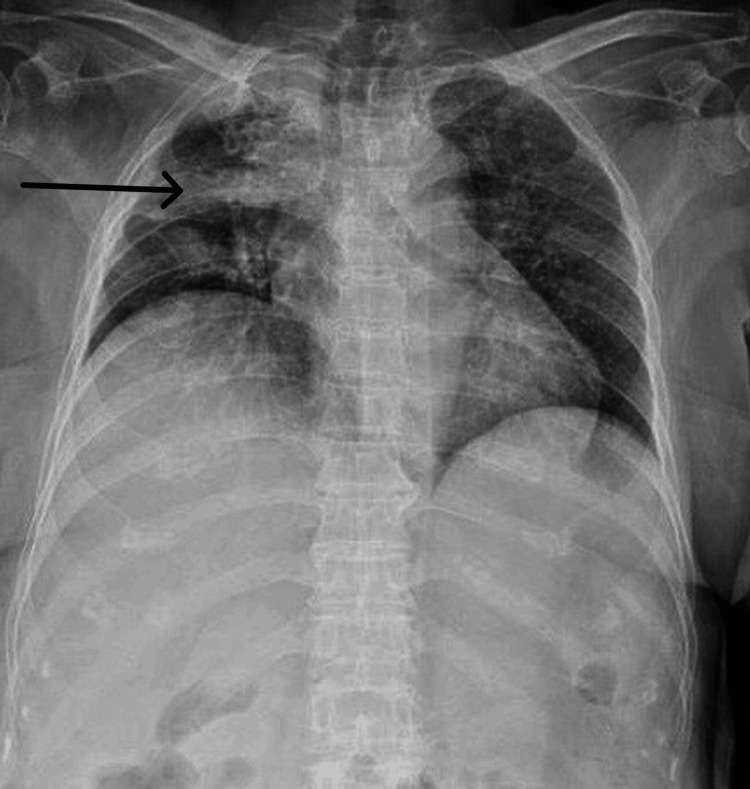
Chest X-ray (posterior-anterior view). Chest X-ray (posterior-anterior view) after treatment showing remarkable improvement in the previously noted right upper lobe consolidation (indicated by the black arrow).

## Discussion

Pulmonary mucormycosis is an aggressive fungal infection associated with high mortality when diagnosis and treatment are delayed. In patients with pneumonia that fails to improve despite appropriate broad-spectrum antibacterial therapy, invasive fungal infections, such as mucormycosis, should be considered, particularly when underlying immunocompromising conditions are present [1].

Mucormycosis is caused by filamentous fungi of the order Mucorales, with *Rhizopus*, *Mucor*, and *Lichtheimia *species being among the most frequently implicated pathogens [4,7]. Pulmonary involvement often presents with non-specific symptoms, including fever, cough, hemoptysis, and pleuritic chest pain, which can resemble a variety of other pulmonary disorders and contribute to delays in diagnosis [8]. The global burden of mucormycosis has risen over recent decades, largely reflecting the growing population of immunocompromised patients. In high-income countries, it is most commonly encountered in individuals with hematologic malignancies or recipients of stem cell transplantation, whereas in low- and middle-income countries, poorly controlled diabetes mellitus remains the predominant predisposing factor [4].

Definitive diagnosis of mucormycosis relies on histopathological demonstration of characteristic fungal elements within affected tissue, ideally supported by microbiological culture. However, cultures are frequently negative, and recognition of broad, ribbon-like, pauciseptate hyphae with right-angle branching on histology may represent the principal diagnostic evidence. Establishing the diagnosis of pulmonary mucormycosis is particularly difficult because its clinical and radiological features overlap substantially with those of other angioinvasive mould infections, bacterial pneumonias, and pulmonary tuberculosis [1,5].

The treatment of mucormycosis requires a comprehensive strategy that includes prompt correction of underlying risk factors, initiation of effective antifungal therapy at appropriate doses, and surgical resection or debridement when feasible. In patients with diabetes mellitus, strict optimization of glycemic control is a critical component of management [9]. Liposomal amphotericin B remains the preferred first-line antifungal agent for pulmonary mucormycosis [10]. Posaconazole and isavuconazole are effective alternatives for patients who are unable to tolerate amphotericin B or as step-down and salvage therapy [11].

When patients demonstrate prompt and sustained clinical improvement, antifungal therapy and supportive measures should be continued. In those who are suitable surgical candidates but fail to show meaningful improvement within 48-72 hours, surgical intervention should be considered. For localized pulmonary mucormycosis, resection of all technically removable infected tissue can play a crucial role in achieving disease control [12]. Outcomes are generally more favorable when the diagnosis is established early, appropriate antifungal therapy is initiated without delay, and complete excision of infected tissue is possible [1].

The prognosis of pulmonary mucormycosis is generally poor because of its aggressive nature; however, survival improves substantially when the infection is recognized promptly and treated early with appropriate antifungal therapy and, when indicated, surgical intervention. Recent advances in diagnostic and therapeutic approaches have the potential to improve outcomes in pulmonary mucormycosis. Molecular techniques, including polymerase chain reaction (PCR)-based assays and next-generation sequencing, allow earlier and more accurate detection of Mucorales compared to conventional culture methods, which frequently yield negative results [13]. Current international guidelines from the European Confederation of Medical Mycology (ECMM) and Mycoses Study Group Education and Research Consortium (MSG ERC) emphasize the importance of early diagnosis, prompt initiation of liposomal amphotericin B, and aggressive correction of underlying predisposing factors to improve survival [1]. Despite these advances, mortality rates remain high, underscoring the need for ongoing research into novel antifungal agents and combination treatment strategies, as well as greater clinical awareness to enable timely diagnosis and intervention [8].

## Conclusions

Pulmonary mucormycosis should be suspected in cases of non-resolving pneumonia, especially in patients with uncontrolled diabetes. Early diagnosis through bronchoscopy and histopathology, followed by prompt antifungal therapy and optimization of underlying risk factors, is essential to improve outcomes and reduce mortality.
